# Is the burden of metastatic lymph node stations a prognostic factor in patients with resected lung cancer?

**DOI:** 10.1186/s13019-024-02874-w

**Published:** 2024-07-03

**Authors:** Necati Çitak, Volkan Erdogu, Yunus Aksoy, Ayşegül Ciftci, Nisa Yildiz, Özgür İsgörücü, Servet Ozdemir, Selçuk Kose, Kenan Can Ceylan, Muzaffer Metin

**Affiliations:** 1 Dr. Suat Seren Chest Disease and Thoracic Surgery Research and Education Hospital, Izmir, Turkey; 2 Yedikule Chest Disease and Thoracic Surgery Research and Education Hospital, Istanbul, Turkey; 3 Bakırkoy Dr. Sadi Konuk Research and Education Hospital, Istanbul, Turkey

**Keywords:** Non-small cell Lung Cancer, Nodal classification, Lymph Node Station, Metastasis, Prognosis

## Abstract

**Objectives:**

The burden of metastatic lymph node (LN) stations might reflect a distinct N subcategory with a more aggressive biology and behaviour than the traditional N classification.

**Methods:**

Between 2008 and 2018, we analyzed 1236 patients with pN1/2 lung cancer. Survival was analyzed based on LN station metastasis, determining the optimal threshold for the number of metastatic LN stations that provided additional prognostic information. N prognostic subgrouping was performed using thresholds for the number of metastatic LN stations with the maximum chi-square log-rank value, and validated at each pT-stage.

**Results:**

Survival showed stepwise statistical deterioration with an increase in the number of metastatic LN stations., Threshold values for the number of metastatic LN stations were determined and N prognostic subgroupswas created as sN-alpha; one LN station metastases (*n* = 632), sN-beta; two-three LN stations metastases (*n* = 505), and sN-gamma; ≥4 LN stations metastasis (*n* = 99). The 5-year survival rate was 57.7% for sN-alpha, 39.2% for sN-beta, and 12.7% for sN-gamma (chi-square log rank = 97.906, *p* < 0.001). A clear tendency of survival deterioration was observed from sN-alpha to sN-gamma in the same pT stage, except for pT4 stage. Multivariate analysis showed that age (*p* < 0.001), sex (*p* = 0.002), tumour histology (*p* < 0.001), IASLC-proposed N subclassification (*p* < 0.001), and sN prognostic subgroups (*p* < 0.001) were independent risk factors for survival.

**Conclusion:**

The burden of metastatic LN stations is an independent prognostic factor for survival in patients with lung cancer. It could provide additional prognostic information to the N classification.

**Supplementary Information:**

The online version contains supplementary material available at 10.1186/s13019-024-02874-w.

## Key question

Is the burden of metastatic lymph node stations a prognostic factor for lung cancer?

## Key findings

Survival rates showed a gradual decline in statistical significance as the number of metastatic lymph node stations incresead.

## Take-home message

The burden of metastatic lymph node stations could provide additional prognostic information to the N classification in patients with lung cancer.

## Introduction

The current N classification for non-small cell lung carcinoma (NSCLC) is based solely on the anatomic location of the involved lymph nodes (LNs). Although the International Association for the Study of Lung Cancer (IASLC) analyses suggested that pN1 and pN2 could be subdivided into N1a, N1b, N2a1, N2a2, and N2b, the recommendation was ultimately deferred because the number of available cases did not allow the validation of each pN category across each pT category [1]. Additionally, the IASLC committee recommends that physicians should record the number of metastatic LNs (or stations) and classify the N category using new descriptors for further testing [1]. Validation studies conducted in line with this recommendation, have yield different results, both supporting and not supporting the proposed N classification [2–6]. Many researchers have investigated the prognostic impact of several factors, such as the number of involved LN zones, number of LNs examined, number of positive LNs, ratio of positive LNs to total dissected LNs, combination of the number and anatomical location of metastatic LNs, and number of involved LN stations [4–6]. However, almost all factors except the number of involved LN stations depend on the skill of the surgeon who removed the LN, experience of the pathologist examining the LNs, annual number of cases in the centre where the surgery was performed, and whether sampling or complete LNs removal was performed. Counting the number of LN station metastases is easier compared to the other methods mentioned above.

This study aimed to evaluate the prognostic significance and discriminatory capability of the number of metastatic LN stations, regardless of the anatomical location. We also examined whether the number of metastatic LN station prognostic categories could be validated across each pT category as per the latest IASLC classification.

## Materials and methods

This study was approved by the Ethics and Scientific Committee of the Bakırköy Sadi Konuk Hospital and was conducted in accordance with the principles of the Declaration of Helsinki. The Institutional Review Board approved the data collection and analysis methods (Decision No: 302-7).

We retrospectively analysed data of 3243 pathological (p) T1-4N1-2M0 patients who underwent resection for NSCLC in two different thoracic surgery departments between 2008 and 2018. We excluded patients without LN metastases (pN0, *n* = 1293), who underwent lung resection after neoadjuvant treatment (*n* = 356), who underwent surgery for recurrent lung cancer (*n* = 155), had incomplete resection (*n* = 99), had fewer than dissected less than three mediastinal LN stations (*n* = 13), who did not have subcarinal LNs (*n* = 12), and those without postoperative follow-up (*n* = 79). The remaining 1236 patients with NSCLC were analysed.

### Preoperative, perioperative and postoperative period

All patients were routinely evaluated using positron emission computed tomography (PET-CT), chest computed tomography (CT), fibreoptic bronchoscopy, and cranial magnetic resonance imaging during the preoperative period. Patients with mediastinal or hilar LNs measuring > 1 cm on chest CT and/or positive PET–CT findings underwent mediastinal staging. Cervical mediastinoscopy was used for mediastinal staging between 2008 and 2013, endobronchial ultrasound-guided transbronchial needle aspiration was performed as a first step in recent years in case of mediastinal staging necessary.

In our centres, systemic mediastinal/hilar LN dissection is performed regardless of the tumour size and location. Almost all mediastinal and hilar LN stations were dissected en bloc, along with the surrounding adipose connective tissue. Intraparenchymal LN stations were removed as distinct nodes to reveal all LNs, and the resected materials and LNs were histopathologically assessed by experienced pathologists. The IASLC nodal map was used to classify the anatomical locations of the LNs [7].

Postoperative adjuvant treatment is administered to patients with stage IIA–IIIB disease, although the oncologist has the option to administer radiotherapy in the presence of chest wall invasion, multilevel N2 disease, extracapsular extension of N2 disease, or mediastinal invasion of a T4 tumour. However, some patients may refuse this treatment or are unable to complete the procedure properly because of side effects.

### Stratification of N status

We performed two different stratifications of LN status assessment: the newly proposed N classification (new-N category) and the number of LN station(s) metastases (station-N category).

The pN status was subcategorised based on the new N as follows: single N1 LN station metastasis (N1a), multiple N1 LN station metastasis (N1b), single N2 LN station metastasis without N1 metastasis (N2a1), single N2 LN station metastasis with N1 metastasis (N2a2), and multiple N2 LN station metastasis (N2b) [1].

Since there were no published studies determining station-N (sN) subcategories thorough statistical method, we established cut-off points for the number of metastatic LN stations. We performed Kaplan-Meier survival analysis based on the number of metastatic LN stations, regardless of the anatomical location. Subsequently, we compared the survival of stations by station (one LN station vs. two LN stations, two LN stations vs. three LN stations, etc.). The cut-off values for sN prognostic classification were determined by the highest chi-square and hazard ratio values in these comparisons with statistically significant survival differences at these breakpoints. The number of sN subcategories was determined by the number of cut-off points providing additional prognostic information, and they were labelled in Greek alphabetical order ; sN-alpha, sN-beta, sN-gamma, sN-delta, etc.

### Statistical analysis

We used the Statistical Package for the Social Sciences software (IBM SPSS Statistics for Windows, Version 23.0; Armonk, NY, USA) for data entry and analysis. Descriptive statistics were used to summarise pertinent study information. Quantitative variables were presented as mean, maximum (max), and minimum (min) values, and qualitative variables were presented as percentage values. The Kolmogorov-Smirnov test was used to examine whether the data were normally distributed. Normally distributed data were reported as mean values. Student’s t-tests were used for comparisons between groups. Pearson’s chi-square test was used to analyse qualitative variables. Non-parametric continuous variables, presented as median values, were compared using the Mann-Whitney U test. Overall survival (OS) was defined as the time between surgery and death. It was estimated using the Kaplan-Meier method and compared between groups using log-rank analysis. Multivariate survival analysis was performed using the Cox proportional hazards model to examine the association between survival and potential prognostic factors. Statistical significance was set at *p* < 0.05.

## Results

Patient characteristics are summarized in Table 1. The most common histopathological tumour type was squamous cell carcinoma (*n* = 694, 56.1%). Lobectomy and pneumonectomy were performed in 807 (65.3%) and 429 (34.7%) patients, respectively. The median tumour diameter was 4.5 cm (min = 0.1 cm, max = 20 cm). The mean number of resected LN stations was 6.2 (min = 5 resected LN stations, max = 10 resected LN stations, median = 6 resected LN stations). According to the IASLC-proposed N classification, 512 patients (41.4%) were pathologically identified as having N1a disease, 271 (21.9%) had N1b, 119 (9.6%) had N2a1, 201 (16.3%) had N2a2, and 133 (10.8%) had N2b disease (783 patients with pN1 disease and 453 with pN2 disease). The mean number of stations with metastasis was 1.7 ± 1.0 (min = 1, max = 7, median = 1). It was 1.4 ± 0.6 in pN1 patients (min = 1 LN station metastasis, max = 4 LN station metastasis, median = 1 LN station metastasis), and 2.4 ± 1.2 in pN2 patients (min = 1 LN station metastasis, max = 7 LN stations metastasis, median = 2 LN stations metastasis). The adjuvant therapy rate was 73.2% (*n* = 905). There were 191 patients who refused adjuvant therapy after surgery. Seventy two patients could not receive treatment due to the distance to the cancer treatment center, and the remaning 68 patients could not receive it, due to comorbidities or advanced age.

**Table 1:** Patients’ characteristics

Variables	Data
**Age, year, median (IQR)**	60 (55–67)
**Sex, n/%**	
**Women** **Men**	155/12.5 1081/87.5
**Cell type n/%**	
**Sqcc** **Ade** **Others**	694/56.1 457/37.0 85/6.9
**Tumour diameter, cm, median (IQR)**	4.5 (3.2-6)
**pT status, n/%**	
**T1a** **T1b** **T1c** **T2a** **T2b** **T3** **T4**	11/0.9 57/4.6 126/10.2 329/26.6 168/13.6 293/23.7 252/20.4
**pN status, n/%**	
**N1a** **N1b** **N2a1** **N2a2** **N2b**	512/41.4 271/21.9 119/9.6 201/16.3 133/10.8
**Resection type, n/%**	
**Lobectomy** **Pneumonectomy**	807/65.3 429/34.7
**Operation type, n/%**	
**VATS** **Open thoracotomy**	91/7.3 1145/92.7
**No. of resected LN stations, mean ± SD**	6.1 ± 1.3
**Surgical mortality, n/%**	37/3.0
**Adjuvant treatment, n/%**	905/73.2

SD, standard deviation; n, number; Sqcc, squamous cell carcinoma; Ade, adenocarcinoma; LN, lymph node; IQR, interquartile range

### Kaplan-Meier survival estimates based on the IASLC proposed N classification (new-N category) and redefined N prognostic subgroups, which were determined by the number of involved LN stations (sN-category)

The 5-year survival rates were 56.0% for pN1 patients (median survival, 73 months) and 29.9% for pN2 patients (median survival, 31 months) (*p* < 0.001). There was a statistically significant difference in survival between N1a and N1b patients (61.6% vs. 45.2%, *p* < 0.001). However, there was no statistically significant difference between N1b and N2a1 patients in terms of survival (45.2% vs. 41.7%, *p* = 0.259). N2a1 patients had a better prognosis than N2a2 patients (41.7% vs. 30.7%), although the difference was not statistically significant (*p* = 0.111). N2a2 patients had significantly better survival rates than N2b patients (35.8% vs. 17.4%, *p* = 0.01) (Supplementary Fig. 1).

The overall survival deteriorated as the number of metastatic LN stations increased (Supplementary Fig. 2). When survival was analyzed per LN station metastasis increment, a progressive decline in survival was observed for each cut-off point (Table 2a). However, some cut-off points were not statistically significant (Table 2b). We observed that two cut-off points for station(s) metastases provided additional prognostic information after considering the other cut-off points in the stepwise selection process. The highest chi-square and HR values in the survival comparisons per LN station metastases were the comparisons between one LN station metastasis and two LN station metastases (chi-square = 17.993, HR = 1.455, 95% CI = 1.210–1.750), and three LN station metastases and four LN station metastases (chi-square = 10.746, HR = 1.668, 95%CI = 1.185–2.348). Only these comparisons were statistically significant in terms of survival (*p* < 0.001 and *p* = 0.001, respectively). Based on these results, the sN prognostic subgroups were created as follows: sN-alpha (sNα), a single LN station metastasis; sN-beta (sNβ), 2–3 LN stations metastasis; and sN-gamma (sNƔ), 4 and above (≥ 4) LN stations metastasis.

**Table 2:** Survival analysis for per lymph node stations metastases and for gradual lymph node station(s) metastasis

*Survival analysis for LN station(s) metastases*						
**Number of metastatic LN station(s)**	No. of patients	MST (months)	5-year survival rate	*P*	Chi-square	HR (%95 CI)
1 versus ≥ 2 LN stations metastases	632 vs. 604	75 vs. 35	57.7% vs. %34.9	**< 0.001**	52.914	1.721 (1.482–1.999)
≤ 2 versus ≥ 3 LN stations metastases	990 vs. 246	63 vs. 26	51.5% vs. 26.9%	**< 0.001**	60.607	1.919 (1.566–2.351)
≤ 3 versus ≥ 4 stations metastases	1137 vs. 99	58 vs. 19	49.6% vs. %12.7	**< 0.001**	70.222	2.545 (1.816–3.565)
≤ 4 versus ≥ 5 LN stations metastases	1213 vs. 23	51 vs. 12	47.2% vs. %6.2	**< 0.001**	23.062	2.822 (1.355–5.877)
***Survival analysis for per LN station(s) metastases***						
**Number of metastatic LN station(s)**	No. of patients	MST (months)	5-year survival rate	*P*	Chi-square	HR (%95CI)
1 versus 2 LN stations metastases	632 vs. 358	75 vs. 40	57.7% vs. 40.6%	**< 0.001** ^**a**^	17.993	1.455 (1.210–1.750)
2 versus 3 LN stations metastases	358 vs. 147	40 vs. 34	40.6% vs. 36.7%	0.09	2.962	1.229 (0.960–1.574)
3 versus 4 LN stations metastases	147 vs. 76	34 vs. 20	36.7% vs. 13.4%	**0.001** ^**a**^	10.746	1.668 (1.185–2.348)
4 versus 5 LN stations metastases	76 vs. 16	20 vs. 12	13.4% vs. 12.5%^#^	0.547	0.362	1.164 (0.689–1.964)
5 versus 6 + 7 LN stations metastases*	16 vs. 7	12 vs. 9	12.5%^#^ vs. 0.0%^#^	0.898	0.016	1.098 (0.882–1.345)

^a^ It was accepted as a cutpoint (threshold) for LN station(s) metastases since these cutpoints had the highest chi-square and HR values. * There were only two patients who had seven different LN station metastases. ^#^ Four-year survival rate; HR, hazard ratio; CI, confidence interval

**Fig. 1:**
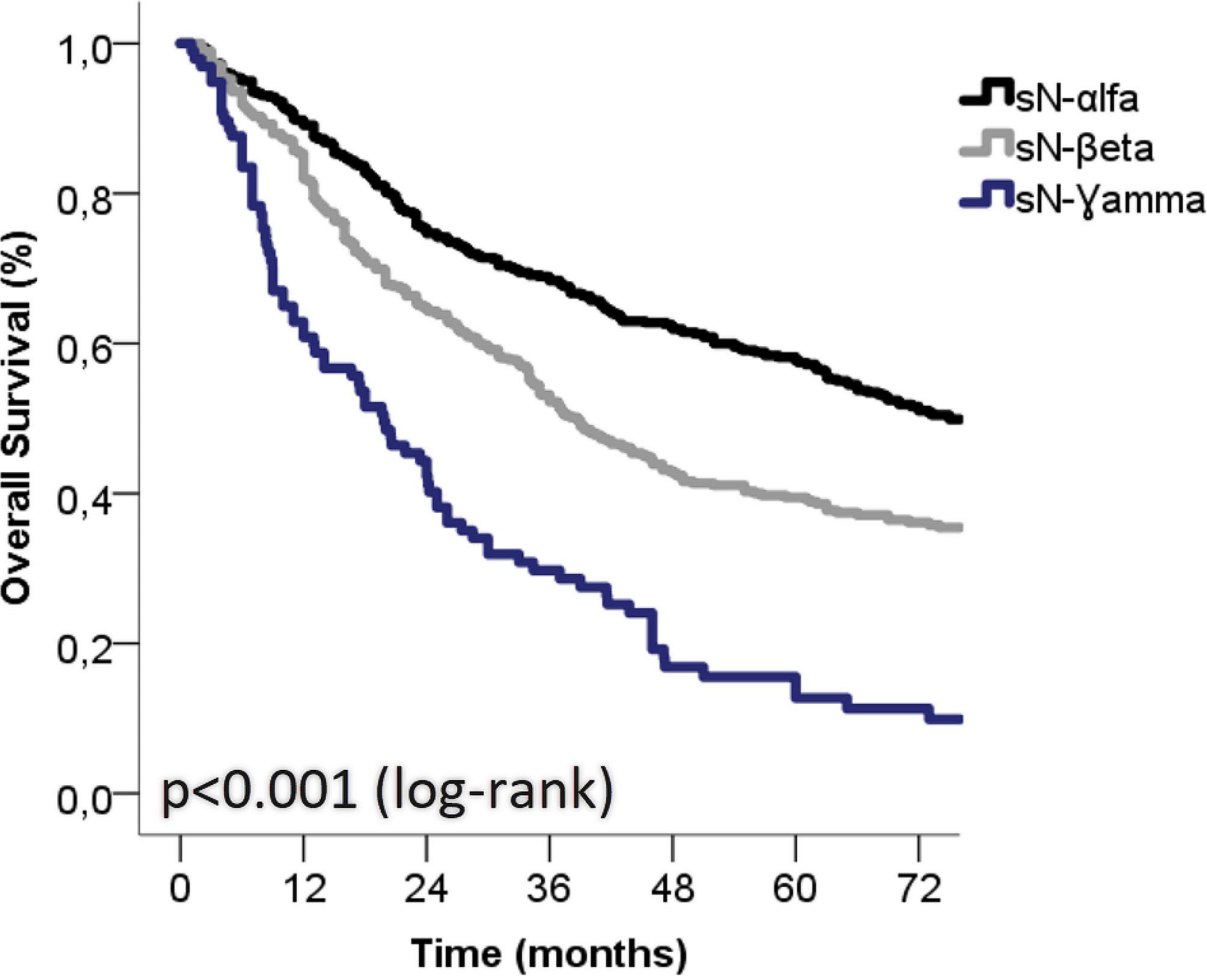
Overall survival curves based on Kaplan-Meier analysis according to the sN subcategories

The survival rates were statistically different among the sN prognostic subgroups and deteriorated from sNα to sNƔ (Fig. 1). The 5-year survival rate was 57.7% for sNα patients (median survival = 75 months), 39.2% for sNβ patients (median survival = 39 months), and 12.7% for sNƔ (median survival = 19 months) (*p* < 0.001, log-rank). The differences in survival between sNα and sNβ and between sNβ and sNƔ were statistically significant (*p* < 0.001, HR = 1.545, 95% CI = 1.315–1.815, and *p* < 0.001, HR = 1.985, 95%CI = 1.465–2.690, respectively).

### Multivariate analyses for survival

Multivariate analysis revealed that age (*p* < 0.001), sex (*p* = 0.002), tumour histology (*p* < 0.001), IASLC-proposed N classification (*p* < 0.001), and sN prognostic subgroups (*p* < 0.001) were independent prognostic factors for overall survival (Table 3).

**Table 3:** Multivariate analyses for overall survival

Variables	Multivariate analysis 1			Multivariate analysis 2		
	HR	95%CI	*p* value	HR	95%CI	*p* value
**Age (per year)**	1.025	1.016–1.034	**< 0.001**	1.020	1.011–1.029	**< 0.001**
**Sex**						
**Women**	Ref			Ref		
**Men**	0.678	0.528–0.871	**0.002**	0.693	0.540–0.891	**0.004**
**Cell type**						
**Non-Adenocarcinoma**	Ref			Ref		
**Adenocarcinoma**	1.253	1.057–1.486	**0.001**	1.346	1.137–1.592	**0.001**
**Tumour diameter (per cm)**	1.019	0.968–1.074	0.473	1.020	0.968–1.076	0.457
**pT status**			0.06			0.06
**T1**	Ref			Ref		
**T2** **T3** **T4**	1.082 1.263 1.352	0.861–1.359 0.975–1.636 1.054–1.734	0.499 0.07 0.01	1.079 1.252 1.327	0.859–1.355 0.968–1.621 1.035–1.702	0.513 0.08 0.02
**pN status**			**< 0.001**			
**N1a**	Ref					
**N1b** **N2a1** **N2a2** **N2b**	1.403 1.830 2.219 3.055	1.138–1.730 1.410–2.376 1.784–2.760 2.400-3.889	**0.04** **< 0.001** **< 0.001** **< 0.001**			
**sN status**						**< 0.001**
**sN-αlfa**				Ref		
**sN-βeta** **sN-**Ɣ**amma**				1.524 3.023	1.297–1.791 2.346–3.894	< 0.001 < 0.001
**Resection type**			0.116			
**Lobectomy**	Ref			Ref		
**Pneumonectomy**	1.149	0.967–1.365		1.087	0.916–1.289	0.338
**No. of resected LN stations (per LN station)**	0.996	0.942–1.053	0.883	0.998	0.950–1.062	0.877
**Adjuvant treatment**						
**No**	Ref			Ref		
**Yes**	0.953	0.802–1.131	0.580	0.960	0.808–1140	0.960

Multivariate analysis 1 for pN categories that was proposed by IASLC, Multivariate analysis 2 for sN prognostic subgroups, pN, pathological-N; N1a, single N1 station metastasis; N1b, multiple N1 stations metastasis; N2a1, pN2 single with skip metastasis; N2a2, pN2 single without skip metastasis; N2b, multiple N2 statios metastasis; sN; classification based on the number of metastatic lymph node stations, sN-αlfa, a single LN station metastasis; sN-βeta, 2–3 LN stations metastasis; and sN-Ɣamma, 4 and above (≥ 4) LN stations metastasis, LN, lymph nodes; pT, pathological tumour stage; HR, hazard ratio; CI, confidence interval

### Internal validation of the sN subcategories with each pT status and cross-validation of the prognostic significance for sN subcategories in N1 and N2 patients

**Fig. 2:**
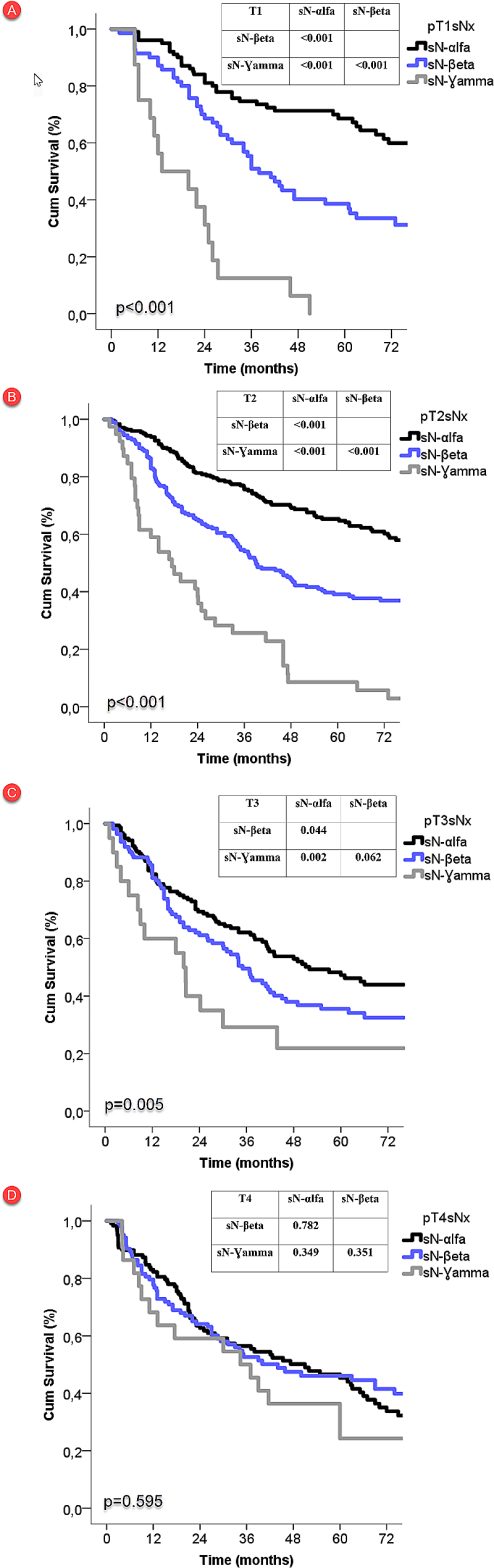
Survival curves according to sN status across each pT stage for internal validation

Internal validation of the sN subcategories in terms of survival was performed for each pT status. We observed a clear tendency of deterioration of survival from sNα to sNƔ within the same pT stage, with the exception of pT4 stage. In the pT4 stage, the differences in survival curves among adjacent sN subcategories decreased (Fig. 2).

Cross-validation of the sN categories for prognostic significance was carried out separately for N1 and N2 patients. When patients with N1 or N2 were divided into sN-α, sN-β, and sN-Ɣ subgroups, statistically significant prognostic differences were observed between each pair of sN subcategories in terms of survival (Supplementary Fig. 3a and 3b).

Conversely, there were notable variations in overall survival between patients with N1 and N2 disease when categorized under the same sN-α and sN-β groups. In sN-α, the 5-year survival rate for pN1 patients was 61.0% whereas it was 41.7% in pN2 patients (*p* < 0.001) (Supplementary Fig. 4a). In sN-β, pN1 patients exhibited a higher survival rate than pN2 patients (46.5% versus 31.4%, *p* = 0.001) (Supplementary Fig. 4b). However, there was no significant difference between patients with N1 and N2 disease when classified under the same sN-Ɣ category (*p* = 0.793). In sN-Ɣ, the 5-year survival rates were nearly identical between N1 and N2 disease (16.7% versus 11.0%) (Supplementary Fig. 4c).

For Stage IIB patients (*n* = 429), the 5-year survival rate was 70.6% in the sN-α category, 48.0% in sN-β, and 0% in sN-Ɣ categories (*p* < 0.001). In Stage IIIA patients (*n* = 616), the 5-year survival rate was 49.6% in sN-α, 35.8% in sN-β, and 6.2% in sN-Ɣ categories (*p* < 0.001). In Stage IIIB patients (*n* = 191), the 5-year survival rate was 35.4% in sN-α, 28.8% in sN-β, and 19.2% in sN-Ɣ categories (*p* = 0.592).

## Discussion

In the 7th edition of the TNM staging system, the IASLC committee suggested that the overall disease burden of LNs might have the most important influence on survival outcomes rather than the anatomical location of LN involvement [7]. Some published studies indicated that the burden of metastatic LN stations might reflect a distinct N-subcategory with more aggressive behaviour than the traditional N-classification, and an increase in the number of involved LNs is a poor prognostic factor [4, 6, 8–11]. Classifications based solely on anatomical location may overlook the tumor burden in LN stations, which is the most distinct limitation of N classification. In the 9th edition, the IASLC committee will focus on the number of LN stations as a new variable for categorising the extent of nodal involvement [9]. Therefore, a count-based staging system will be necessary. Furthermore, the use of different LN maps, variations in interpretations between N1 and N2 by different surgeons, and the number of stations resected may impact the final staging [12, 13]. In the most recent IASLC database, there was a significant variation in 5-year survival rates among pN2 cases from America, Asia, and Europe (42%, 39%, and 22%, respectively) [1]. The use of different LN maps, due to the retrospective nature of the submitted data may explain these survival differences. Therefore, an N definition independent of anatomical localization, focusing solely on the number of involved LNs, may provide clarity. In the present study, we developed a prognostic LN classification that regards the anatomical distinction between N1 and N2 instead emphasizing the number of metastatic LN stations. We found that the number of positive LN stations is a strong independent prognostic factor for lung cancer.

Although it is clear that there is a difference in survival between patients with single and multiple (either N1 or N2) LN station diseases [14, 15], the number of published studies with a sufficient number of patients on the relationship between the number of metastatic LN stations and lung cancer survival is very small [4, 11]. Similar to our study, Xu et al. [4] stated in their study consisting of 1249 cases that sN classification is a more effective survival predictor than the IASLC-proposed N classification. In this study, the cases were grouped according to the number of metastatic LNs: metastasis to a single LN, metastasis to two LNs, and metastasis to three or more LNs. Notably, the pN2 population in this study was significantly higher than that in the present study (71% vs. 36.6%). Despite this difference, it can be assumed that regardless of the number of pN2 patients and geographical differences, sN classification successfully predicts prognostic outcomes.

Kojima et al. [11] stated in their comprehensive study including 405 patients (211 in pN1, 194 in pN2) that although the IASLC-proposed N classification is a good prognostic indicator for predicting survival outcomes, they found overlaps in survival rates. Therefore excluding anatomic localization, they grouped the cases similar to our study as Nα (1 LN station metastasis), Nβ (2–3 LN station metastasis), and NƔ (4 + LN station metastasis) and observed statistically significant differences in survival. They stated that this is a more accurate prognostic indicator than the IASLC-proposed N classification and suggested that it might not be necessary to consider the boundary between N1 and N2. Although similar cut-off points for sN subcategories were used in previous studies, the cut-off points of the station based on the nodal category have never been determined with statistical analyses. In the present study, we set thresholds according to the statistical analyses of station-based classification, which differs from other studies.

The number of involved LN stations regardless of anatomical location in N classification showed a clear tendency of deterioration of survival from sN-αlfa to sN-Ɣ in the same pT stage except in pT4 stage. This situation showed that the sN category results were validated across each T stage, except for pT4. In the pT4 stage, the differences in survival curves among neighbouring sN subcategories diminished. These results can be observed in IASLC studies and demonstrate that the prognostic value of N in patients with T4 patients deteriorates [1, 6].

In contrast, there were significant differences in overall survival between patients with N1 and N2 disease when staging in the same sN categories except sN-Ɣ. For example, the survival outcome of a patient with a positive LN at station 4R was different from that of a patient with a positive LN at station 12 (in sN-αlfa). Similarly, the survival outcome of a patient with a positive LN at station 4R-7-11 was different from that of a patient with a positive LN at station 10-11-12 positive LNs stations (in sN-βeta). As mentioned in our previous studies, the importance of anatomical localization in LN metastasis has been demonstrated in many different studies [6, 8, 16]. Therefore, although sN plays a role as a prognostic indicator in multivariate analyses, discarding classifications based on anatomical location is not possible.

The sN categories may facilitate the assessment of the effect of adjuvant treatment, could allow more aggressive adjuvant therapies and require strict follow-up in patients with more LN station metastases compared to patients with fewer LN station metastases. There is still not enough data to present regarding its potential benefits according to the number of metastatic LN stations, which may better define an indication for adjuvant treatment. In a recent meta-analysis, it was reported that the association between a higher metastatic LN ratio (the number of metastatic LN / the number of total dissected LN) and the benefit of adjuvant treatments [17]. In our study, there was a clear tendency of deterioration of survival from sN-αlfa to sN-Ɣamma in the same stages of lung cancer. This finding might suggest that lung cancer patients in the same stage with different sN level descriptors should receive different postoperative adjuvant therapies. sN stratification may be particularly important in patients with N1 disease or at stage IIB where schedules and indications for adjuvant therapies are still being debated and may lead to the addition of adjuvant radiotherapy to treatment in pN2 patients with high levels of the burden of the metastatic LN station [18, 19]. It was shown that postoperative radiotherapy did not improve survival times of lung cancer patients with pN2-low metastatic LN density but significantly improved survival times of pN2-high metastatic LN density [20].

The recently published 9th TNM proposal for lung cancer suggests subdividing N2 into subgroups N2a and N2b based on the number of metastatic mediastinal lymph nodes [21]. This reflects the IASLC committee’s recognition of the importance of metastatic lymph node burden in N2 disease, as supported by our study findings. The reclassification of T1N2a to Stage IIB in the 9th TNM proposal represents the most significant change in the TNM classification [22]. However, altering the treatment approach for T1N2a tumors should await new prospective studies and should align with clinical guidelines for Stage IIIA cancers. This principle also applies to T1/2sN-alpha, which exhibited the best prognosis in our study (with 5-year survival rates of 68% and 64%, respectively). As emphasized by the IASLC committee, changes in anatomical classification should not automatically dictate changes in treatment [22]. Therefore, recommendations for upfront surgery or surgery after neoadjuvant therapy in patients clinically diagnosed with T1N2a or T1/2sN2-alpha should be based on appropriately designed clinical trials.

### Limitations

The present study had several limitations. First, it was a retrospective study. Second, we could not standardize lymphadenectomy; however, we excluded patients with inadequate LN station dissection. Although our study included data from experienced centres that used standardized procedures for every step, it should be noted that LN station labeling may vary between surgeons. Third, alternative approaches regarding N status must achieve greater prognosis discriminatory ability, be clinically coherent, and be backward compatible with the incumbent staging system. While the sN prognostic subgroups derived from pathologically staged tumours achieved greater prognosis discriminatory ability, they could not be validated with clinical staging, and counting individual LNs in radiological studies might be too challenging. Therefore, the present findings cannot be applied to nonsurgical patients.

On the other hand, our study had several strengths. It included a sufficient number of patients to describe the LN tumour burden. To the best of our knowledge, this is the second study to report the highest number of patients in the literature.

## Conclusion

The newly proposed N descriptors are appropriate for stratifying heterogeneous patients with N1 and N2 disease into prognostically different subgroups. The number of positive LN stations (tumour burden of LN stations) is a novel prognostic indicator in patients with lung cancer and may add different prognostic information to the N classification. However, it has lost its importance in terms of prognosis in anatomical localization-specific comparisons and has not been clinically confirmed. Therefore future multicentre studies are needed for its confirmation.

### Electronic supplementary material

Below is the link to the electronic supplementary material.

**Supplementary Fig. 1**. Overall survival curves based on Kaplan-Meier analysis according to the new-N subclassification

**Supplementary Fig. 2**. Overall survival curves according to the number of metastatic lymph node station(s)

**Supplementary Fig. 3.** (a) Survival curves according to sN subgroups in pN1 patients, (b) Survival curves according to sN subgroups in pN2 patients

**Supplementary Fig. 4**. (a) Survival curves according to pN subgroups in sN-alfa patients, (b) Survival curves according to pN subgroups in sN-beta patients, (c) Survival curves according to pN subgroups in sN-Ɣamma patients

## Data Availability

The data presented in this article cannot be shared publicly because of the privacy of the study participants. However, the data will be shared upon reasonable request from the corresponding author.

## Abbreviations

CI: Confidence interval
HR: Hazard ratio
IASLC: International Association for the Study of Lung Cancer
LNs: Lymph nodes
NSCLC: Non-small cell lung cancer
sN: N category based on the number of metastatic stations
