# Supplementary figures and images for: Is the burden of metastatic lymph node stations a prognostic factor in patients with resected lung cancer?
Source: J Cardiothorac Surg. 2024 Jul 3;19:413. doi: 10.1186/s13019-024-02874-w (PMC11221125; doi:10.1186/s13019-024-02874-w)

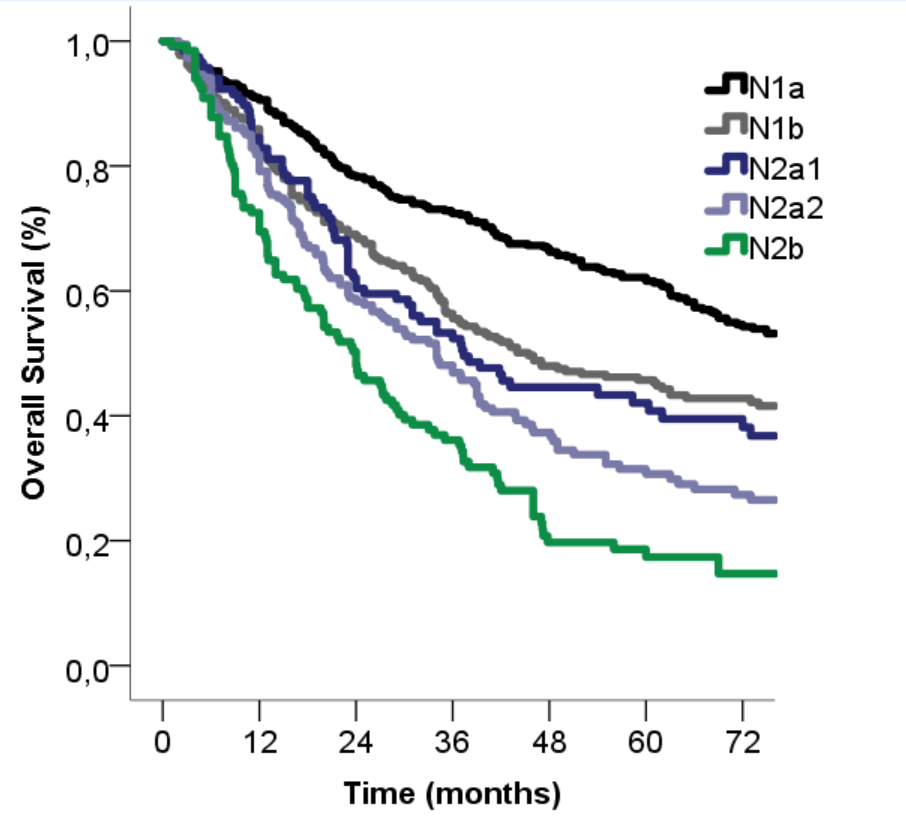

Supplement: Supplementary file 1 — Supplementary Fig. 1. Overall survival curves based on Kaplan-Meier analysis according to the new-N subclassification [file 13019_2024_2874_MOESM1_ESM.tif]

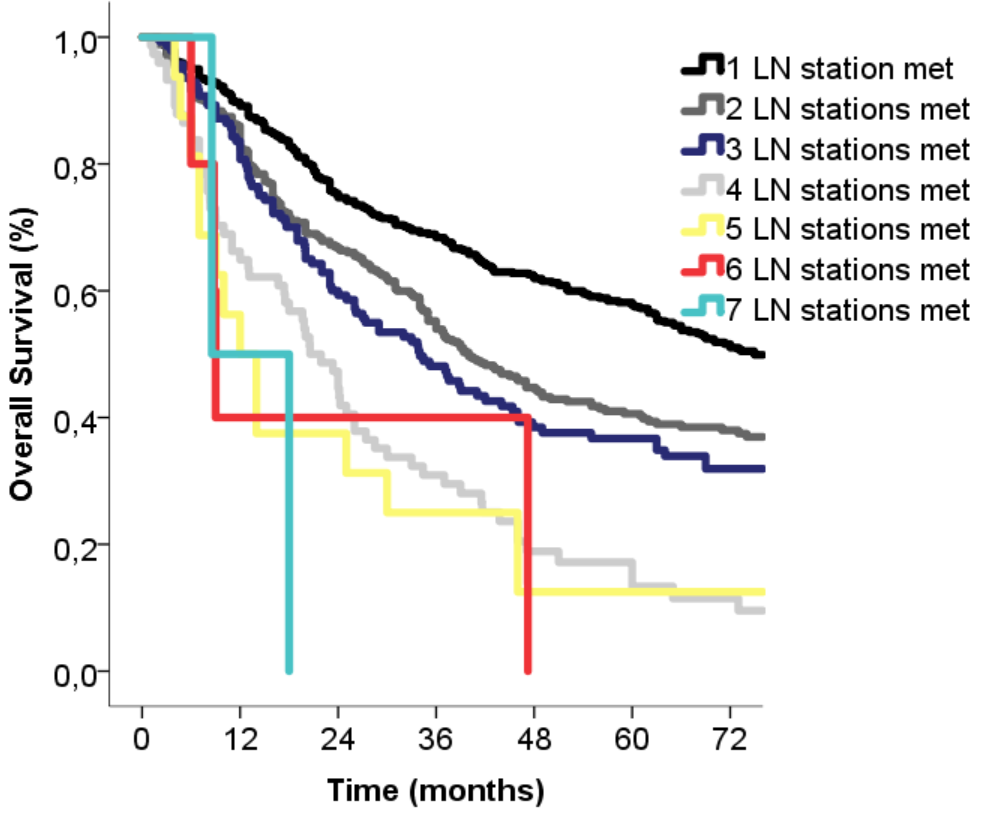

Supplement: Supplementary file 2 — Supplementary Fig. 2. Overall survival curves according to the number of metastatic lymph node station(s) [file 13019_2024_2874_MOESM2_ESM.tif]

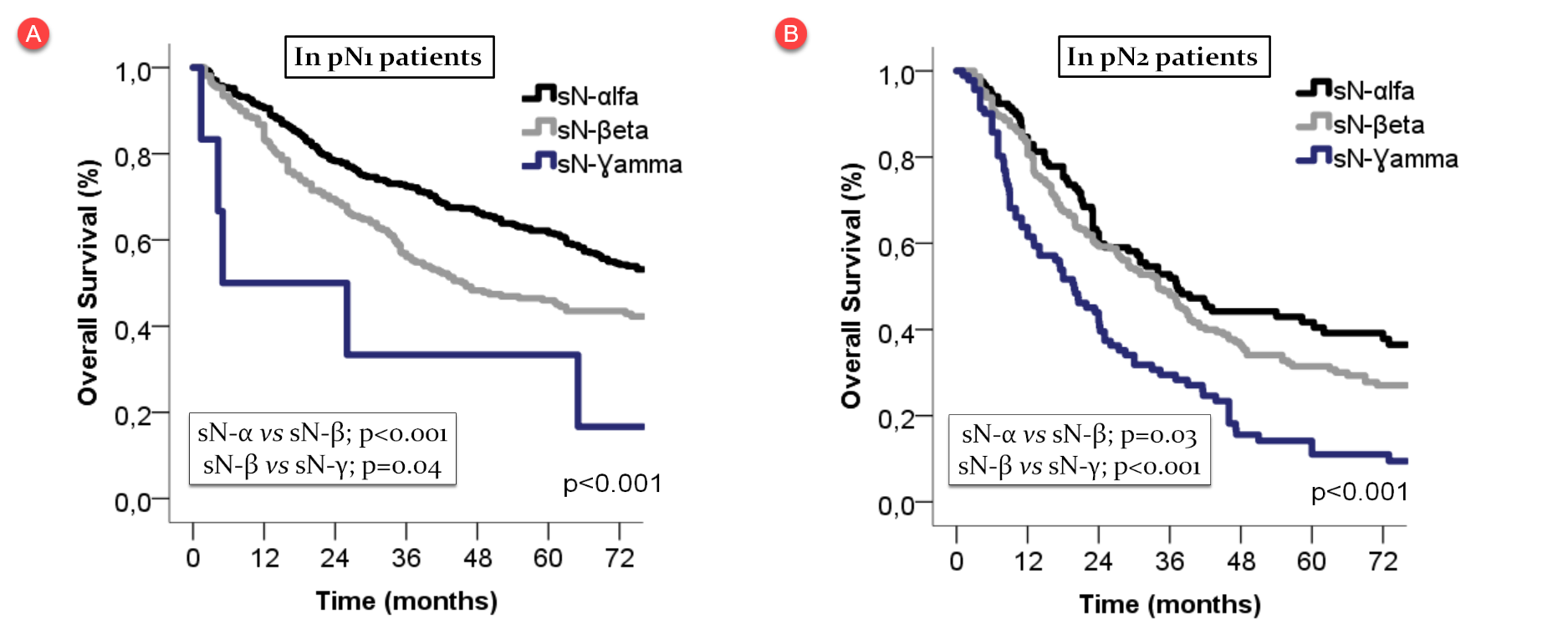

Supplement: Supplementary file 3 — Supplementary Fig. 3. (a) Survival curves according to sN subgroups in pN1 patients, (b) Survival curves according to sN subgroups in pN2 patients [file 13019_2024_2874_MOESM3_ESM.tif]

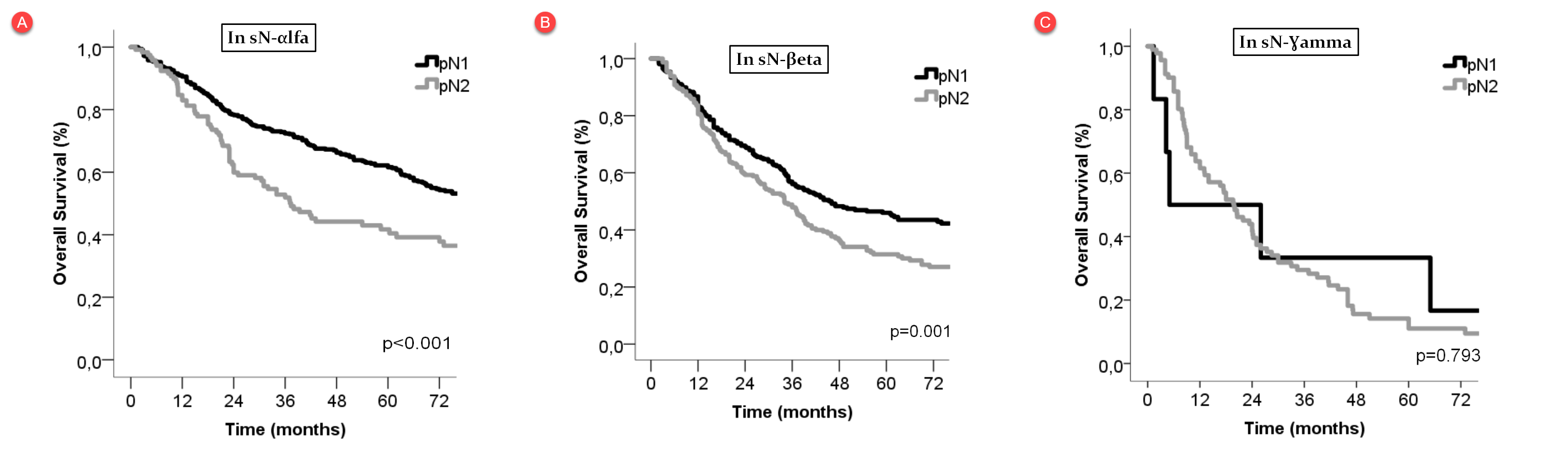

Supplement: Supplementary file 4 — Supplementary Fig. 4. (a) Survival curves according to pN subgroups in sN-alfa patients, (b) Survival curves according to pN subgroups in sN-beta patients, (c) Survival curves according to pN subgroups in sN-Ɣamma patients [file 13019_2024_2874_MOESM4_ESM.tif]
